# Proteomics and Metabolomics Unveil *Codonopsis pilosula* (Franch.) Nannf. Ameliorates Gastric Precancerous Lesions *via* Regulating Energy Metabolism

**DOI:** 10.3389/fphar.2022.933096

**Published:** 2022-07-19

**Authors:** Rupu He, Ruyun Ma, Zheng Jin, Yanning Zhu, Fude Yang, Fangdi Hu, Jianye Dai

**Affiliations:** ^1^ School of Pharmacy, Lanzhou University, Lanzhou, China; ^2^ School of Pharmacy, Gansu University of Chinese Medicine, Lanzhou, China; ^3^ Collaborative Innovation Center for Northwestern Chinese Medicine, Lanzhou University, Lanzhou, China

**Keywords:** proteomics, metabolomics, gastric precancerous lesions, energy metabolism, *Codonopsis Radix*

## Abstract

**Objective:** This study aimed to systematically evaluate the efficacy of *Codonopsis pilosula* (Franch.) Nannf. (*Codonopsis Radix*, CR) and reveal the mechanism of its effects on suppressing Gastric Precancerous Lesions.

**Methods:** First, we established the GPL rat model which was induced by N-methyl-N′-nitro-N-nitrosoguanidine, a disordered diet, and 40% ethanol. The CR’s anti-Gastric Precancerous Lesions effect was comprehensively evaluated by body weight, pathological section, and serum biochemical indexes. Then, quantitative proteomics and metabolomics were conducted to unveil the disturbed protein-network and pharmacodynamic mechanism. Furthermore, serum pharmacology was employed to confirm that CR’s anti-gastritis and anti-cancer phenotype in cell models.

**Results:** In animal models, CR had been shown to control inflammation and ameliorate Gastric Precancerous Lesions. Considering the combination of proteomics and metabolomics, we found that CR could significantly reverse the biological pathways related to energy metabolism which were disturbed by the Gastric Precancerous Lesions model. Furthermore, the results of serum pharmacology indicated that the *Codonopsis Radix* containing serum could ameliorate gastritis injury and selectively inhibit the proliferation of gastric cancer cells rather than normal cells, which was closely related to ATP production in the above mentioned cells.

**Conclusion:** In summary, CR exerted anti-Gastric Precancerous Lesions effects by ameliorating gastritis injury and selectively inhibiting the proliferation of gastric cancer cells rather than normal cells. Proteomics and metabolomics unveiled that its efficacy was closely related to its regulation of the energy-metabolism pathway. This research not only provided new ideas for exploring the mechanism of complex systems such as Chinese herbals but also benefited the treatment strategy of Gastric Precancerous Lesions via regulating energy metabolism.

## Introduction

The 2020 World Health Organization (WHO) Cancer Report shows that gastric cancer is one of the most common cancers in the world, with the fifth highest morbidity rate and the fourth highest mortality rate in malignant tumors (https://www.who.int/data/gho/publications/world-health-statistics). Surgery and chemotherapy currently are the main treatments for gastric cancer. However, the 5-year survival rate of these patients is still less than 30% even after surgical intervention, due to the high possibility of recurrence and metastasis (Zong et al., 2016). Commonly chemotherapy for gastric cancer includes fluorouracil, doxorubicin, and platinum compounds (Kolarić et al., 1986; Al-Batran et al., 2016; Patel et al., 2021). Yet, due to their inevitable drug resistance and toxic side effects, patients have poor compliance and prognosis.

According to the Correa model (Correa et al., 1975), the occurrence of gastric cancer often goes through the following process: normal gastric mucosa, inflammation, atrophy, metaplasia, dysplasia, and gastric cancer. Among them, atrophy, intestinal metaplasia, and dysplasia have been classified as precancerous lesions. Chronic inflammation is a crucial factor in this process (Maeda et al., 2001; Wang et al., 2014; Deswaerte et al., 2018; Duarte et al., 2018). However, it is often difficult for patients to cooperate because of mild symptoms. And it is too late to block “tumor deterioration”. Therefore, it is most feasible to block or reverse the “inflammation-cancer transformation” process. Through the “preventive treatment” strategy, the goal of reducing the incidence rate and the mortality rate of gastric cancer is expected to be achieved.

In fact, “preventive treatment” is one of the core concepts of traditional Chinese medicine. In clinical practice, many traditional prescriptions have been applied, such as the Sijunzi decoction (Zhong et al., 1997; Cai et al., 2008), Xiangshaliujunzi decoction (Lv et al., 2017; Hong et al., 2020), etc. Among these decoctions, *Codonopsis pilosula* (Franch.) Nannf. [Campanulaceae] (*Codonopsis Radix*, *Codonopsis pilosula*, Dangshen in Chinese, CR) is the principal herbal. It has been found that the polysaccharide of CR has anti-ulcer and anti-tumor pharmacological effects (Yang and Zhou, 2022). In addition, lobetyolin of CR, a polyacetylene compound, has a good protective effect on gastric mucosal damage caused by ethanol and has obvious anti-ulcer effects (Song et al., 2008; Bailly, 2021). Modern pharmacology has proved that CR and its active ingredients can treat stomach diseases (Sui et al., 2005; Xu et al., 2008; Li et al., 2017; Bailly, 2021) by ameliorating gastrointestinal motility (Wang et al., 1997; Wang et al., 2015) and regulating oxidase levels (Ma et al., 2014; Li et al., 2017). However, the detailed molecular mechanism is still unclear.

Therefore, we aim to systematically evaluate the pharmacodynamic effects of CR in GPL animal models. Furthermore, the potential mechanism and targeted pathways will be explained by proteomics and metabolomics. Finally, we will confirm our hypothesis via cellular models. It is expected to provide new revelations for the treatment of GPL and explore the mechanism of *Codonopsis pilosula* (Franch.) Nannf., a tonifying-spleen herbal.

## Materials and Methods

### Materials and Reagents

*Codonopsis pilosula* (Franch.) Nannf. collected from Weiyuan County (Gansu Province, China), which was identified as *Codonopsis pilosula* (Franch.) Nannf. by Prof. Xicang Yang from the Affiliated Hospital of the Gansu University of Chinese Medicine. The main components of CR are polysaccharides (40–50%) and oligosaccharides (10–20%), and their extraction and component analysis have been reported previously (Chunxia et al., 2013; Bai et al., 2020). Vitacoenzyme was purchased from Deshengtang Pharmacy (Lanzhou, China). N-methyl-N′-nitro-N-nitrosoguanidine (M105583) was purchased from Aladdin (Shanghai, China). Ethyl ether (2020061501) was purchased from Kelong (Tianjin, China). CCK8 kit (M4839) was purchased from Abmole (TX, United States). Triton X-100 (T8787), Triethylammonium bicarbonate buffer (18597, TEAB), Urea (U5378), DL-Dithiothreitol (43815, DTT), Iodoacetamide (I1149, IAA), CH_2_O (F1635), CD_2_O (492620), ^13^CD_2_O (596388), NaBH_3_CN (42077), NaBD_3_CN (190020), ammonium hydroxide solution (221228), formic acid (F0507) were purchased from Sigma (MI, United States). BCA protein assay kit (23225), Methanol (A456-4), Acetonitrile (A955-4), Isopropanol (A461-4) were purchased from Thermo Fisher Scientific (MA, United States). Trypsin (V5280) was purchased from Promega (WI, United States). ATP Test Kit (ADS-W-A001-96) was purchased from Kexing (Shanghai, China). Anti-Caspase three antibody (ER30804) and Anti-Caspase 12 antibody (ER62907) were purchased from HUABIO (Hangzhou, China). An anti-NF-κB antibody (8242 S) was purchased from CST (BSN, United States). Anti-β-actin antibody (SA00001-1) was purchased from Proteintech (CHI, United States). All metabolite standards were purchased from Sigma (MI, United States), Steraloids (Beijing, China), and TRC Chemicals (YTO, Canada). Human gastric mucosal cells (GES-1 cells) and Human gastric carcinoma cells (AGS cells) were purchased from China Center for Type Culture Collection (Wuhan, China).

To prepare the water extract, 3 kg CR were soaked in 24L water for 30 min and boiled for 1 h at 100°C. Then, the first extract was filtrated, and then 12 L water was added to decoct for the second extract. After the combination, the extracts were concentrated at 0.36 kg/L.

### Animal Experiments and Sample Collection

Male Wistar rats (6 weeks old, average weight 220 g) were purchased from Changsheng Biotechnology of Liaoning province in China (SCXK- 2015–0001), raised in the Animal Experiment Center of Gansu University of Chinese Medicine (50–70% humidity, 23–25°C, 12–12 h light/dark cycle). After 7 days of acclimatization, rats were randomly divided into seven groups, including Control group (*n* = 12, Control), Control group treated with high-dose of CR (*n* = 6, Control + CR(H)), GPL Model group (*n* = 12, Model), low-dose of CR group (*n* = 6, CR(L)), middle-dose of CR group (n = 6, CR(M)), high-dose of CR group (*n* = 6, CR(H)), and positive control group (*n* = 6, P-Control).

According to the reported protocol (Kim et al., 1999), a high-concentration of MNNG (0.2 g/kg/15 days) was given by gavage, combined with a disordered diet (1 time/3 days) and alcohol drinking (40% ethanol gavage, 1 ml/3 days), to establish GPL model. Saline was given to the control group and model group. Vitacoenzyme (Zhang and Wang, 2019; Xia et al., 2021) was given to the positive group at 0.28 g/kg/d. And intragastric administration was respectively performed with 0.6, 1.2, and 2.4 g/kg/d intragastrically, once a day for 16 weeks as the low, medium, and high dose of CR groups. All administered dosages were converted according to the clinically effective dose. When all animals were euthanized, blood and gastric tissues were collected. Gastric tissues from the control group, model group, and medium-dose of CR group were used for proteomic and metabolomic experiments.

### Histopathology and Biochemical Index Test

The gastric tissues were fixed in 10% formalin. After dehydrating, the biopsies embedded in wax were sectioned at 3 μm, and stained with hematoxylin and eosin for histopathological examination by light microscopy. Serum biochemical indicators were detected by an automatic biochemical analyzer.

### Stable Isotope Dimethyl Labeling

Trypsin digestion: The rat gastric tissue was rinsed in ice-cold saline to remove the blood, then weighed 200 mg. The tissue was ground for 90 s with a tissue grinder, and the supernatant was collected after centrifuging (21500g, 4°C, 5 min). The protein was diluted to 3 mg/ml after quantification. Ten μL protein diluent was reacted with 30 μL urea (8M, TEAB) and 2 μL DTT (200mM, H_2_O) for 15min incubating at 65 °C in darkness. After the tube cooled to 35°C, 2 μL IAA (400mM, H_2_O) was added for 30 min at 35°C. Then 2 μL DTT (200mM, H_2_O) was added for 15 min incubating at 65°C in darkness. In the end, 100 μL TEAB and 2 μL trypsin (0.2 μg/μL) were added to incubate overnight at 37°C.

Isotope dimethyl labeling: After digestion, the peptides were reacted with 6 μL of CH_2_O (4%) and 6 μL of NaBH_3_CN (0.6M) according to Table 1, and incubated with a constant temperature shaker (800rpm, 22°C) for 1 h. It was stopped by adding 24 μL ammonia solution (1%, v/v) and 12 μL formic acid. Finally, the light, middle, and heavy groups were mixed.

**TABLE 1:** Isotope label grouping table.

Group	CH_2_O	NaBH_3_CN
Health group-light	CH_2_O	NaBH_3_CN
Model group-middle	CD_2_O	NaBH_3_CN
CR-treated group-heavy	^13^CD_2_O	NaBD_3_CN

Desalting: Operating according to the instructions of PierceTM C18 Tips (Thermo Scientific, 87782).

### LC-MS/MS and Data Analysis

The isotope-labeled quantitative proteomics biological mass spectrometry detection and analysis were based on the published technical scheme (Boersema et al., 2009), and were carried out by the Q-Exactive Orbitrap mass spectrometer coupled to the Ultimate 3000 LC system. In short, the flow rate was set to 0.3 μL/min, and the applied remote spray voltage was set to 2.8 kV. Labeled peptide samples were loaded onto a 100 μm fused silica column packed with 15 cm × 3 μm C18 resin. A full scan (350–1,800 MW) was used, followed by data-dependent MS2 scans that enabled dynamic exclusion of the 20 most abundant ions for MS2 data collection. ProLuCID software was used to analyze the LC-MS/MS data. The parameter settings included static modification of cysteine ​​residues (+57.0215 Da) and variable oxidation of methionine residues (+15.9949 Da). The data was further filtered through DTASelect 2.0.4769, and the peptides were restricted to those that were completely digested by trypsin, with a false discovery rate of 1%. According to the published technical scheme (Benjamin et al., 2012), the internal software CIMAGE is used to quantify the ratio of quantitative whole proteomics experiments. Proteins with an average ratio of greater than 1.5 or less than 0.66 in all samples were selected for KEGG pathway analysis and GO analysis by DAVID (https://david.ncifcrf.gov/). The selected proteins were imported into the STRING (http://stringdb.org/) database to construct the protein-protein interaction (PPI) network.

The mass spectrometry proteomics data have been deposited to the ProteomeXchange Consortium via the PRIDE (Perez-Riverol et al., 2022) partner repository with the dataset identifier PXD032714.

### Quantitative Metabonomic Analysis

A total of 10 mg of gastric tissue was homogenized by 20 μL of deionized water. Then, the derivation process was performed as in the previous reports (Xie et al., 2021). Ultra-performance liquid chromatography coupled to a tandem mass spectrometry (UPLC-MS/MS) system was used to quantitate all targeted metabolites in this project. The ultra-performance liquid chromatography coupled to tandem mass spectrometry (UPLC-MS/MS) system (ACQUITY UPLC-Xevo TQ-S, Waters Corp., Milford, MA, United States ) was used to quantitate all targeted metabolites in this project. The column in the metabolomics analysis was ACQUITY UPLC BEH C18 1.7 µM VanGuard pre-column (2.1 × 5 mm). The UPLC-MS/MS operating condition was adapted from previous reports (Xie et al., 2021). To diminish analytical bias within the entire analytical process, the samples were analyzed in the group but the groups were analyzed randomly. The raw data files generated by UP LC-MS/MS were processed using the MassLynx 4.1 software to perform peak integration, calibration, and quantitation for each metabolite. The different metabolites of each group were imported into KEGG (https://www.kegg.jp/) and MBRole 2.0 (http://csbg.cnb.csic.es/mbrole2/) to obtain the relevant metabolic pathways.

### Preparation of Drug-Containing Serum

The blood of rats in each group was collected after intragastric administration according to the dose, centrifuged in a high-speed centrifuge for 10 min (4°C, 12000r/min), the supernatant was taken, and 4 times the volume of absolute ethanol was added to the serum to precipitate the protein. The protein suspension was centrifuged for 10 min (4°C, 15000r/min) to take the supernatant and spin dry (45°C, 1600r/min, 300min) in a vacuum spin dryer.

### Cell Culture

GES-1 cells were cultured in Dulbecco’s modified Eagle medium with 10% FBS and 1% penicillin-streptomycin at 37°C and 5% CO_2_. The cells were inoculated into 96-well plates at 5×10^3^ cells per well and waited for 12 h to adhere. Then the cells were treated with drug-containing serum for 24 h. The culture methods and conditions of AGS cells were the same as that of GES-1 cells.

### CCK8 Assay

The cell viability was tested via a CCK8 kit. After the cells were treated for 20 h, 10 μL of CCK8 reagent was added to each well and incubated for 4 h. The optical density (OD) was detected at 450 nm to measure the cell viability.

### ATP Assay

The cells were collected and disrupted by ultrasonic waves on ice. Then, the samples were centrifuged (12,000 rpm, 4°C) for 10 min, and the supernatant was collected for testing. Ten μL sample was added to the 96-well plates. The chromogenic reagents were added to react with the sample for 5 min at 25 C. The optical density (OD) at 350 nm was measured by a microplate reader and calculated. The concentrations of proteins were detected by the BCA kit.

### Western Blotting

The proteins were separated by electrophoresis in 10% sodium dodecyl sulfate-polyacrylamide gel, and then the separated proteins were transferred to the polyvinylidene fluoride (PVDF) membrane. The PVDF membranes were combined with anti-Caspase three antibody, anti-Caspase 12 antibody, anti-NF-κB antibody, anti-IL-17A antibody, or anti-β-actin antibody incubated overnight at 4 °C, then shaken with secondary antibody at room temperature for 1 h. Exposure was taken in a TANON gel imager.

### Statistical Analysis

Differences between experimental and control groups were determined by unpaired *t*-test or by two-way ANOVA analysis where more than two groups of data were compared. *p* values <0.05 were considered statistically significant. Graphs were prepared in Prism software (GraphPad, La Jolla, CA).

## Results

### *Codonopsis pilosula* (Franch.) Nannf. Could Significantly Ameliorate Gastric Precancerous Lesions in the MNNG-Induced Animal Model

**FIGURE 1:**
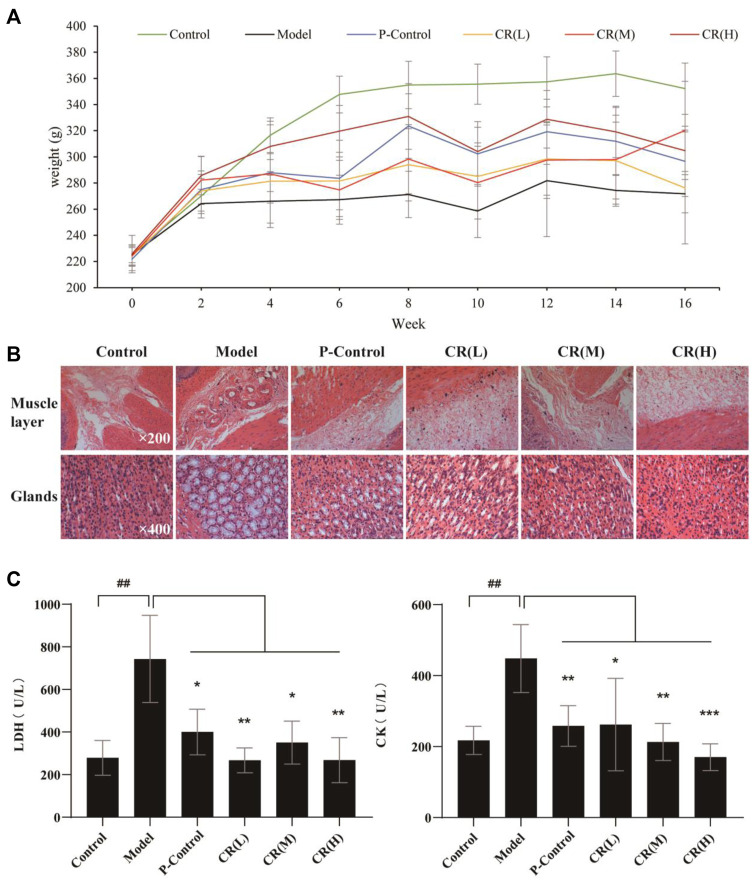
The pharmacodynamic results of CR in the treatment of GPL rats. **(A)** Weight diversity curve of animal experiments. **(B)** HE stained pathological section of gastric tissue in different group. **(C)** The contents of LDH (Lactate Dehydrogenase) and CK (Creatine Kinase) in serum of different group. The graphs show the mean ± SD of at five independent experiments (*n* = 5). ^##^ *p* < 0.01 vs Control. ^*^ *p* < 0.05, ^**^ *p* < 0.01 and ^***^ *p* < 0.001 vs. Model.

On the premise of clinical effectiveness, to further determine the therapeutic effects of CR, we adapted N-methyl-N′-nitro-N-nitrosoguanidine (MNNG)-induced GPL model, combined with a disordered diet and alcohol drinking (Kim et al., 1999). As the recorded body weight for 16 weeks, three doses of CR was higher than the model group after the fourth week. Among them, the high-dose group was always higher than the other dose groups, indicating that the state of the rats in the high-dose group might be better than in other models (Figure 1A). Then, the histological results evaluated the improvement of GPL. We observed that inflammatory cell infiltration and increased mast cells were seen in the muscle layer in the model group, which was significantly reduced after administration of CR (Figure 1B). The gastric glands in the model group were loosely arranged and a large number of intestinal metaplasia goblet cells were seen, while that was effectively recovered in the CR group (Figure 1B). Moreover, we found that the biomarkers, LDH and CK, were significantly reduced in CR groups, especially the high-dose group (Figure 1C). And CR presents mild pharmacodynamic effects in SOD and MDA. Supplementary Figure S1C). In addition, the results of healthy rats after administration of CR illustrated that it had few side effects (Supplementary Figures S1A–C). All the above evidence supported that CR was effective and low-toxicity for GPL treatment with long-term administration.

### Quantitative Proteomics Revealed the Pharmacodynamic Network and Mechanism of *Codonopsis pilosula* (Franch.) Nannf.

**FIGURE 2:**
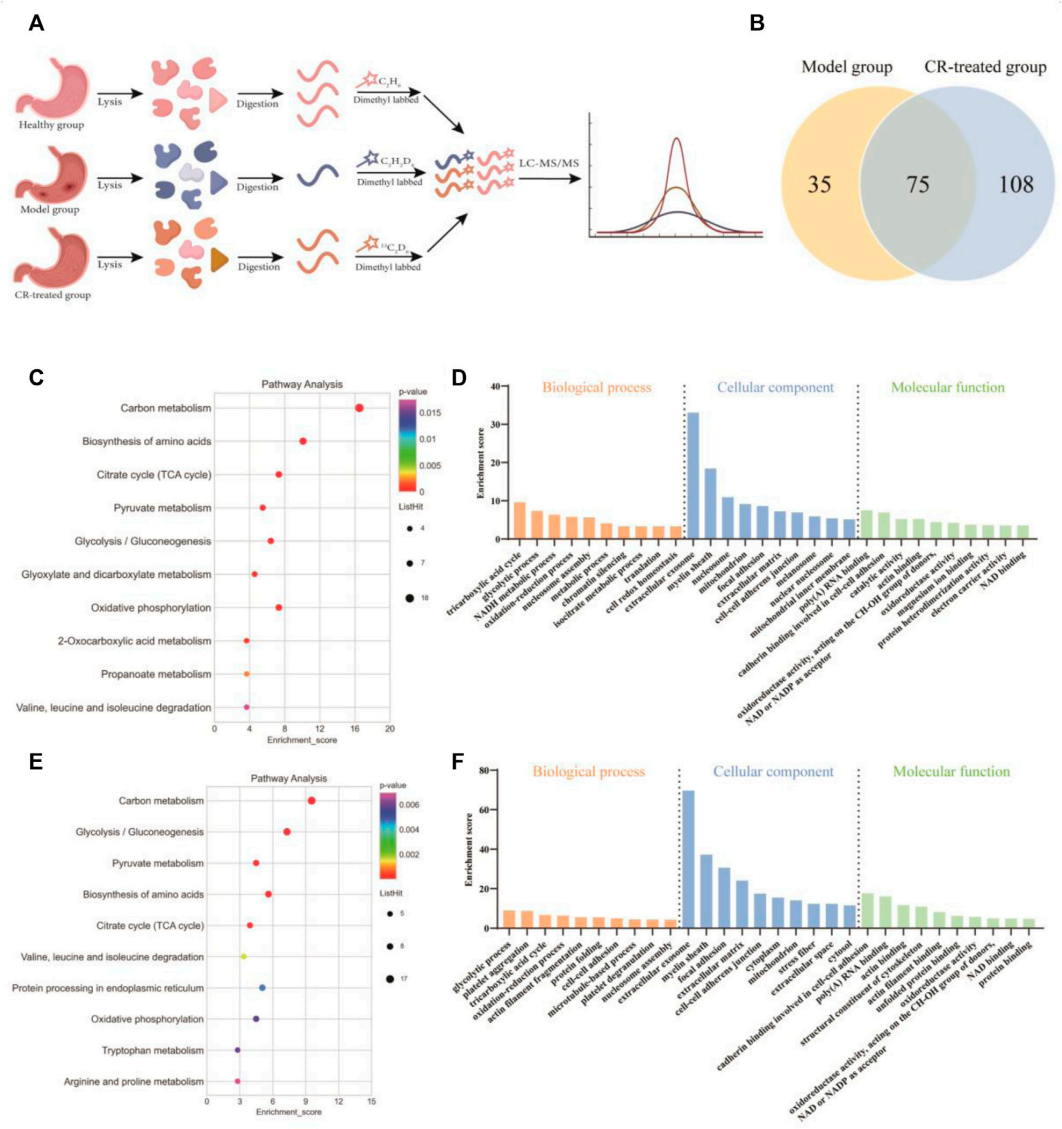
Quantitative proteomics analysis of CR. **(A)** Schematic diagram of quantitative proteomics experiments. **(B)** Venn diagram showing the number of intervened proteins in the model group and CR-treated group. **(C,D)** Analysis of KEGG pathway and biological function (model group vs. healthy group) based on quantitative proteomics. **(E,F)** Analysis of KEGG pathway and biological function (CR-treated group vs. model group) based on quantitative proteomics.

We next employed quantitative proteomics based on stable isotope dimethyl labeling to simultaneously compare the protein networks in three groups (Healthy, model, and CR-treatment) (Figure 2A). After applying a cutoff of the ratio greater than 1.5 or less than 0.66 in all of the model and CR-treated groups, we collectively identified 110 up-regulated proteins in the model group and 183 down-regulated proteins in the CR-treated group as key targets (Figure 2B). According to KEGG and GO analysis, the results showed that the pathways intervened by modeling (Figures 2C,D) were reversed by CR-treatment (Figures 2E, F). Moreover, energy metabolism, such as the citrate cycle and glycolysis process, attracted our attention. In addition, we constructed the protein-protein interaction (PPI) network (Supplementary Figures S2A, B), which indicated that most of the proteins with high degree values were related to the glycolysis process and citrate cycle. Taken together, we hypothesized that CR suppressed the GPL mainly by regulating the pathways of energy metabolism.

### Quantitative Metabolomics Verified That Energy Metabolism Was Critical for the Pharmacodynamic Effect of *Codonopsis pilosula* (Franch.) Nannf.

**FIGURE 3:**
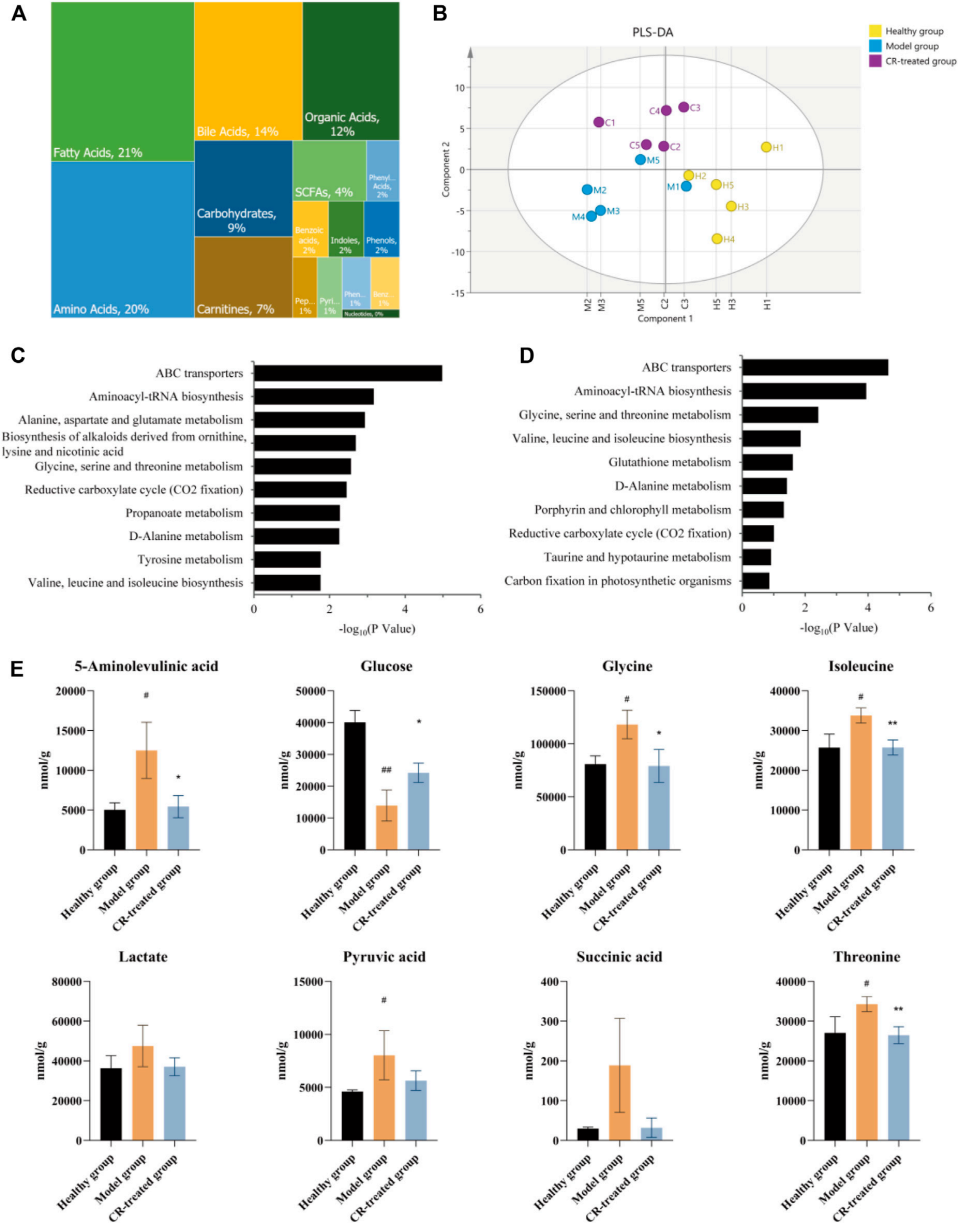
Quantitative metabolomics analysis of CR. **(A)** Relative abundance of each metabolite class in 221 metabolites. **(B)** PLS-DA analysis in healthy group, model group and CR-treated group (*n* = 5). **(C)** Analysis of KEGG pathway (model group vs. healthy group) based on quantitative metabolomics. **(D)** Analysis of KEGG pathway (CR-treated group vs. model group) based on quantitative metabolomics. **(E)** CR intervention reverses the disturbed biomarkers of glycolysis and citrate cycle in GPL animal model. The graphs show the mean ± SD in three independent experiments (*n* = 5). ^#^ *p* < 0.05 vs Healthy group. ^##^ *p* < 0.01 vs Healthy group. *p < 0.05 and **p < 0.01 vs Model group.

To verify whether energy metabolism was critical for the pharmacodynamic effect of CR, quantitative metabolomics was employed to unveil the intervened metabolites after CR treatment. We identified and quantified 221 metabolites in these three groups, of which fatty acids, amino acids, and bile acids were the main metabolites (Figure 3A). Furthermore, PLS-DA analysis was performed on the overall gastric tissue metabolic profile of the healthy group, modeling group, and CR-treated group (Figure 3B). According to the results, the three groups could be well distinguished, and there was a tendency to return to health after CR administration in GPL modeling.

Based on the results of proteomics, we presumed that CR’s efficiency may be derived from the regulation of energy metabolism. Further metabolic analysis could provide evidence for this hypothesis. KEGG analyses revealed the top ten pathways. The metabolic pathway up-regulated in the model group which almost was down-regulated by CR, such as glycine, serine, and threonine metabolism, valine, leucine, and isoleucine biosynthesis, etc. (Figures 3C–E). After sorting out these metabolic pathways, we found that they are mainly some metabolism and synthesis pathways of amino acids, which were highly related to glycolysis/gluconeogenesis and the citrate cycle. In summary, we believed that these amino acids were involved in the carcinogenesis of gastric epithelial cells as the material basis of energy metabolism.

### Serum Pharmacology Confirmed That *Codonopsis pilosula* (Franch.) Nannf. Exerted Significant Anti-Inflammatory and Anti-Gastric-Cancer Effects in Inflammatory and Gastric Cancer Cell Lines

**FIGURE 4:**
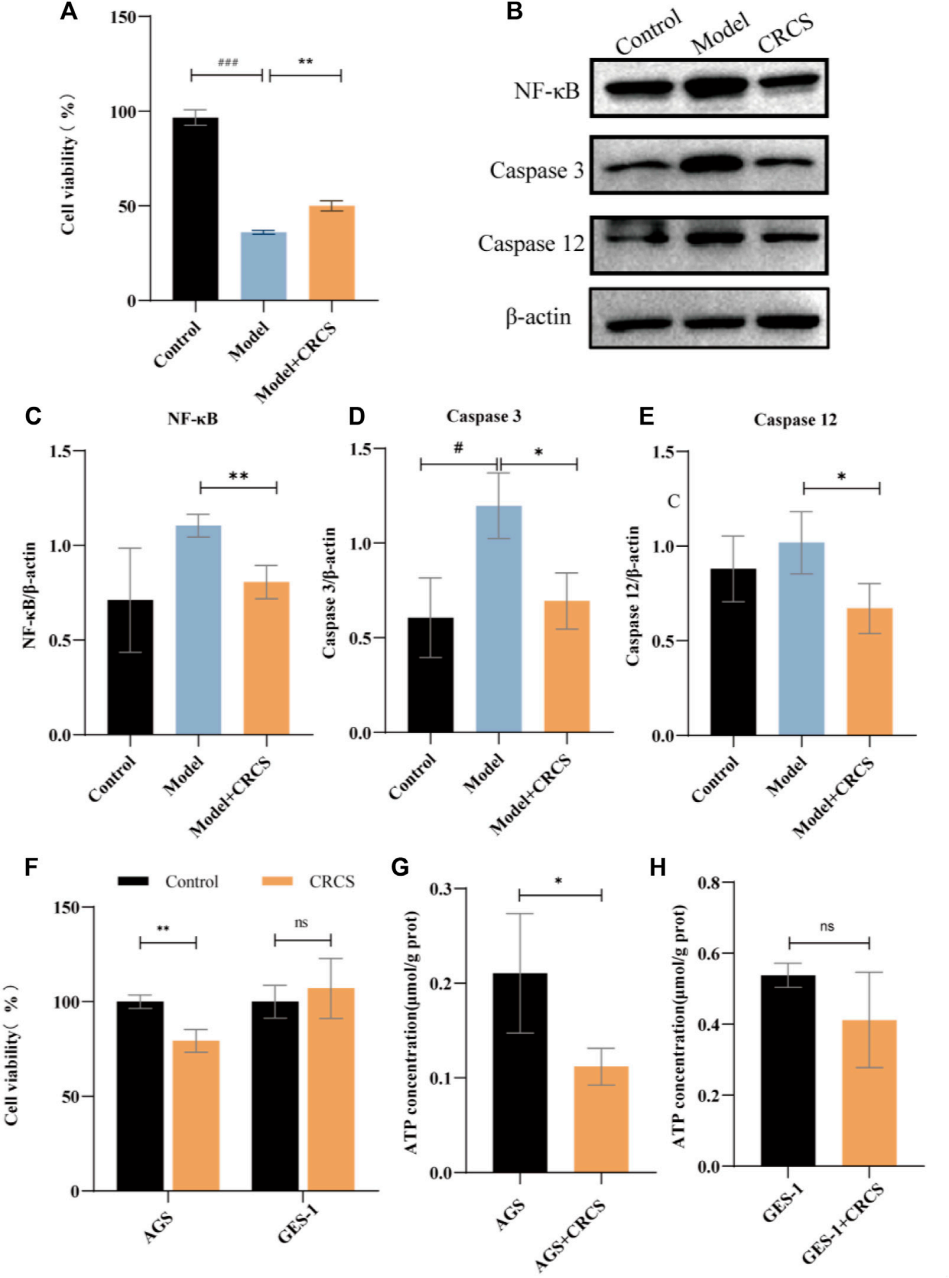
CRCS’s anti-inflammatory and anti-gastric-cancer effect on MNNG-induced GES-1 cells and AGS cells. **(A)** MTS assay of CRCS on MNNG-induced inflammatory GES-1 cells. **(B)** Western blots of inflammatory factors (NF-κB) and apoptosis factors (Caspase three and Caspase 12) in GES-1 cells (Control) and inflammatory GES-1 cells induced by MNNG (Model). **(C)** Western blotting analysis of NF-κB in inflammatory GES-1 cells treated with CRCS. **(D)** Western blotting analysis of Caspase three in inflammatory GES-1 cells treated with CRCS. **(E)** Western blotting analysis of Caspase nine in inflammatory GES-1 cells treated with CRCS. **(F)** MTS assay of CRCS’s effects on AGS and GES-1 cells. **(G)** ATP contents in AGS cells treated with CRCS or not. **(H)** ATP contents in GES-1 cells treated with CRCS or not. β-actin served as the internal control in all Western blots. Data are presented as the mean ± SD, *p < 0.05, **p < 0.01, ***p < 0.001, *n* = 3 per group.

In order to further verify our hypothesis, we conducted gastritis cell models by MNNG-induced normal gastric epithelial cells (GES-1 cells) and gastric cancer cells (AGS) to simulate the possible anti-inflammatory and anti-cancer processes. And serum pharmacology was employed to illustrate CR’s cellular phenotype. Our hypothesis was that the active ingredients of CR need to enter the blood to be effective, and CR-containing serum (CRCS) could act directly on relevant gastric cells. The results showed that CRCS had a protective effect on MNNG-induced inflammatory GES-1 cells (Figure 4A). Then western blot experiments were performed on MNNG-induced inflammatory GES-1 cells and the administration group (Figure 4B). As shown in quantification analysis, it could be seen that higher expression of NF-κB protein in the model group could remarkably reduce after administration of CRCS (Figure 4C). Moreover, the intracellular apoptotic proteins in the model group were increased, such as Caspase-3 and Caspase-12, while those in the administration group were significantly decreased. (Figures 4D,E). In addition, CRCS presented inhibiting effect on gastric cancer cells (AGS cells), yet it was mild for normal gastric epithelial cells (GES-1 cells) (Figure 4F). So, we found the selective inhibiting effect of CRCS, which was consistent with animal experiments.

To further verify our hypothesis that CR could block the transformation of gastric cancer by reducing energy metabolism, we assayed ATP contents before and after administration of CRCS. The results showed that CRCS significantly reduced the energy in AGS cells but had little effect on GES-1 cells (Figures 4G, H). The above results are consistent with our results obtained by proteomics and metabolomics, indicating that regulating energy metabolism is an important way to prevent GPL.

## Discussion

Gastric cancer is the most important gastrointestinal cancer, and its high morbidity and mortality rate seriously endanger human health. At present, the main treatments for gastric cancer still present some shortcomings (Zong et al., 2016). Through an overview of the occurrence process of gastric cancer, we believe that blocking GPL may reduce the incidence and mortality of gastric cancer. Modern pharmacological research has shown that the pharmacological effects of *Codonopsis pilosula* (Franch.) Nannf. cover all stages of gastric cancer and precancerous lesions (Sui et al., 2005; Xu et al., 2008; Li et al., 2017; Bailly, 2021).

In this study, we employed traditional pharmacodynamics and serum pharmacology to evaluate the anti-GPL effects of CR. And quantitative proteomics and metabolomics were conducted to illustrate the mechanisms. First, we established a GPL rat model by MNNG combined with a disordered diet and alcohol drinking. The results of pathological analyses proved that CR could significantly block the progress of GPL. Interestingly, in this study, the aggregation of a small number of mast cells was observed through HE-stained sections, and the number decreased after treatment with CR. Mast cells have been thought to be closely related to allergic reactions since they were discovered by Paul Ehrlich in 1878 (da Silva et al., 2014). Studies have shown that mast cells are related to the release of VEGF, FGF-2, TGF-β, IL-8, and Ang-1 (Crivellato et al., 2004). These angiogenesis-related factors could play a role in all stages of angiogenesis, including degradation of extracellular matrix, migration and proliferation of endothelial cells, and formation and distribution of new blood vessels (Patricia and Robert, 1987). Reducing mast cell aggregation might inhibit angiogenesis in tumors and chronic inflammation, which may be the potential pharmacological process.

According to the results of serum biochemical indicators, CR significantly regulated lactate dehydrogenase (LDH) and creatine kinase (CK). LDH is one of the rate-limiting enzymes in the carbohydrate metabolism pathway. LDH converts pyruvate into lactic acid during glycolysis, which is secreted out of the body by cells, and its increased content helps cancer cells obtain nutrients for growth and division through aerobic glycolysis (Koppenol et al., 2011; Xu et al., 2015). Based on the experimental results, we speculated that CR may reduce the content of LDH to promote the pyruvate of gastric cancer cells and other rapidly proliferating cells to enter the citrate cycle and reduce the production of lactic acid to increase the PH in the tumor microenvironment, which is not conducive to tumor cell invasion. Since CK mainly exists in the cytoplasm and mitochondria, it can affect the cell cycle process by regulating intracellular energy metabolism, which is closely related to the ATP homeostasis of cells (Zhou et al., 2016). However, the energy metabolism in GPL rats was in a compensatory active state, and the CK level is significantly increased. CR might regulate energy metabolism by inhibiting the aerobic glycolysis pathway of gastric cancer cells and fast-proliferating cells related to precancerous lesions. Therefore, we speculated that CR’s anti-GPL efficacy was mainly related to the regulation of energy metabolism.

Protein is the carrier of cell or body function which reflects the process of function change. Metabolite is the final product of body function which reflects the result of function change. Proteomics and metabolomics have been widely used in the study of the pharmacodynamic mechanism by observing the changes in overall protein profile and metabolic profile at the system level and understanding the process and results of functional changes in organisms. To verify our hypothesis, quantitative proteomics and metabolomics were conducted to illustrate that GPL down-regulated energy metabolism, which might in turn affect the synthesis and metabolism of a variety of amino acids (Figure 2F; Figure 3D; Supplementary Figure S3). In addition, with serum pharmacology, we further verified that CR inhibited the proliferation of cancer cells via regulating energy metabolism (Figures 4F–H).

The proliferation of cancerous cells are closely related to glutamine and three other amino acids (glycine, serine, and methionine). Some studies have shown that glutamine intake is increased in many cancers such as pancreatic cancer, ovarian cancer, and breast cancer (Fan et al., 2013; Yang et al., 2014; van Geldermalsen et al., 2016). This was also confirmed in clinical that plasma glutamine concentration of patients with different tumors is significantly lower than that of healthy subjects (Hamberger et al., 1991; Bode and Souba, 1999). Glycine and serine are involved in the synthesis and the conversion of one-carbon units. The one-carbon unit produces nucleotides, proteins, and lipids through the cycle of folate and methionine, which is an important source of substances for tumor cell growth (Locasale, 2013). In our metabolomic results, a variety of amino acid metabolism and synthesis pathways in the model group were up-regulated, including the synthesis materials of glutamine such as glutamic acid and isoleucine, the metabolism products of glutamine such as glutamic acid, aspartic acid, and pyruvate, the source of the one-carbon unit such as glycine and serine. It revealed that the metabolic cycle of glutamine and the formation of the one-carbon unit were closely related to the GPL model rats. In addition, it could also be seen in the proteomics results that CR had a certain regulatory effect on the synthesis and decomposition of various amino acids (Figures 2E, F). Therefore, we speculated that CR could inhibit the metabolic cycle of glutamine and the formation of the one-carbon unit, which reduces the growth of tumor cells.

Through the proteomics and metabolomics research on gastric tissue, we believe that the cancerous cells have changed the energy metabolism-related pathways in order to grow and invade rapidly, preferring aerobic glycolysis to obtain the required energy and substances, resulting in the up-regulation of multiple amino acid metabolism and synthesis. CR down-regulates the related pathways of these amino acids and reduces their content, which may relate with treat GPL through energy metabolism.

## Conclusion

**FIGURE 5:**
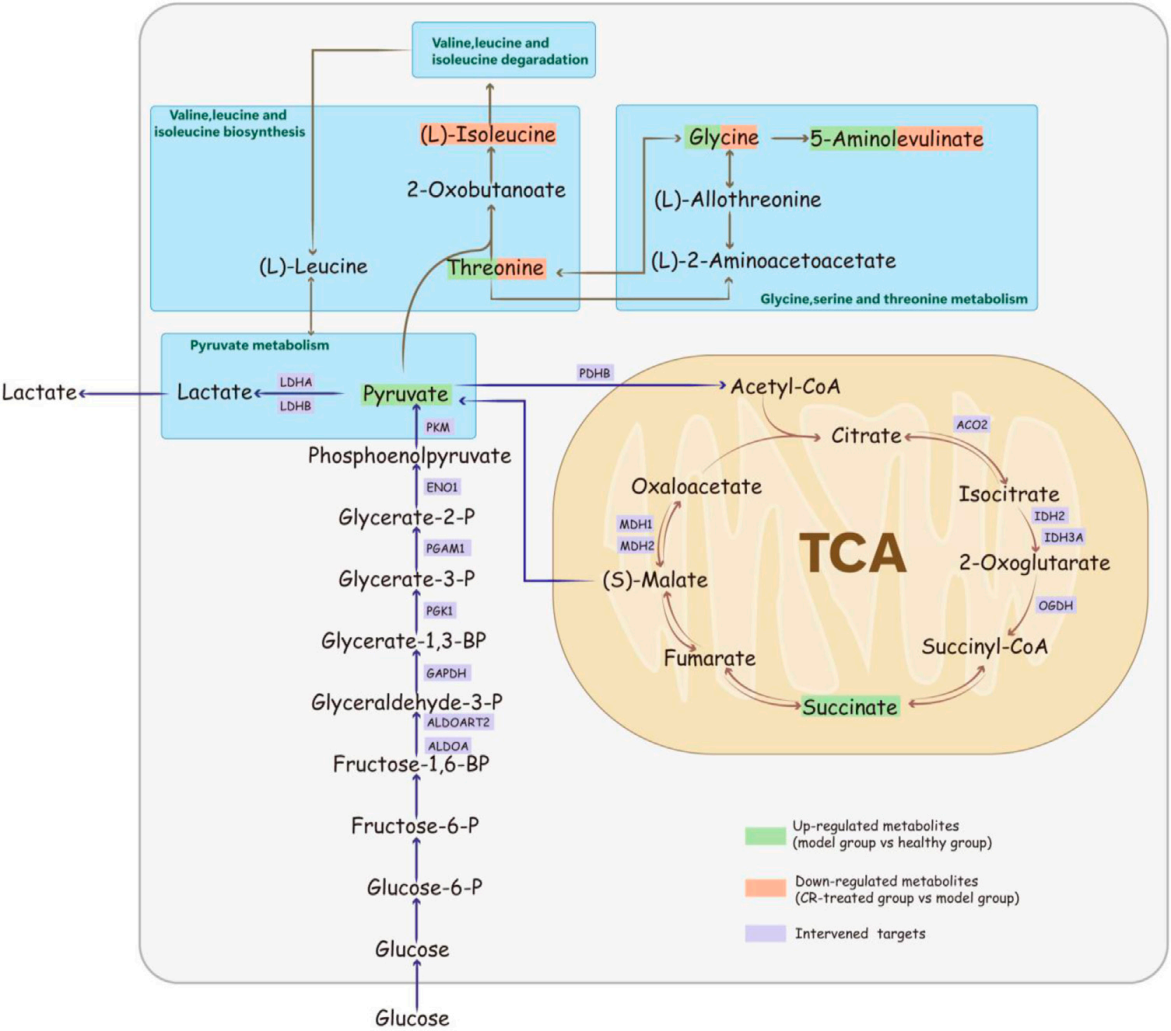
Schematic diagram of *Codonopsis pilosula* (Franch.) Nannf. ‘s mechanism for suppressing GPL via the energy-related pathway.

In this study, we conducted a rat model to evaluate the anti-GPL effects of CR. Combining the proteomic and metabolomic analysis, we claimed that CR mainly through the regulation of glycolysis and tricarboxylic acid cycle and other processes, thereby affecting the energy metabolism of cancer cells to block the transformation of gastric cancer (Figure 5). In addition, we directly verified CR’s anti-inflammatory and select tumor-inhibiting effects, which were closely related to cellular ATP. Thus, we claimed that CR could ameliorate inflammation and apoptosis via regulating energy metabolism pathways. This observation showed the pharmacodynamic mechanism of CR in suppressing GPL, which may provide a new perspective for exploring the mechanism of herbal medicine, especially traditional Chinese medicine. Regulating energy metabolism can provide a new strategy for early intervention of gastric cancer.

## Data Availability

The original contributions presented in the study are included in the article/Supplementary Material; further inquiries can be directed to the corresponding authors.

## Abbreviations

CR, Codonopsis pilosula (Franch.) Nannf; GPL, Gastric Precancerous Lesions; CRCS, Codonopsis pilosula (Franch.) Nannf. containing serum; MNNG, N-methyl-N′-nitro-N-nitrosoguanidine; KEGG, Kyoto Encyclopedia of Genes and Genomes; GO, Gene Ontology.
