# Supplementary material for: Proteomics and Metabolomics Unveil Codonopsis pilosula (Franch.) Nannf. Ameliorates Gastric Precancerous Lesions via Regulating Energy Metabolism
Source: Front Pharmacol. 2022 Jul 19;13:933096. doi: 10.3389/fphar.2022.933096 (PMC9343858; doi:10.3389/fphar.2022.933096)
Supplement: Supplementary file 1 [file DataSheet1.docx]

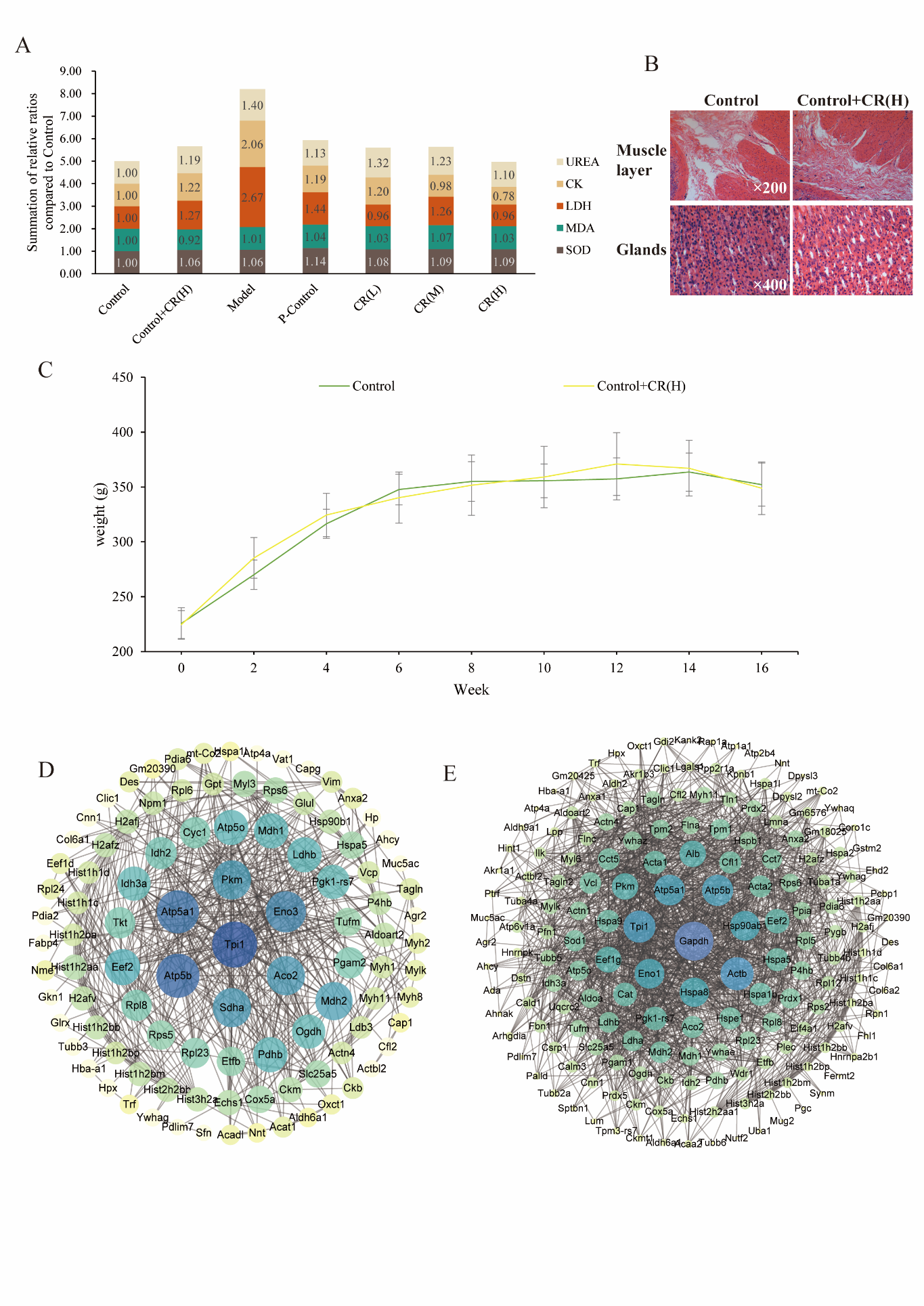


**Figure S1 |** Effects of CR(H) on weight and biomarkers of GPL rats. **(A)** The contents of serum biochemical index in different group. **(B)** HE stained pathological section of gastric tissue in CR(H) group. **(C)** Weight diversity curve in CR(H) group. **(D-E)** The model group/healthy group PPI(D) and the experimental group/model group PPI (E).
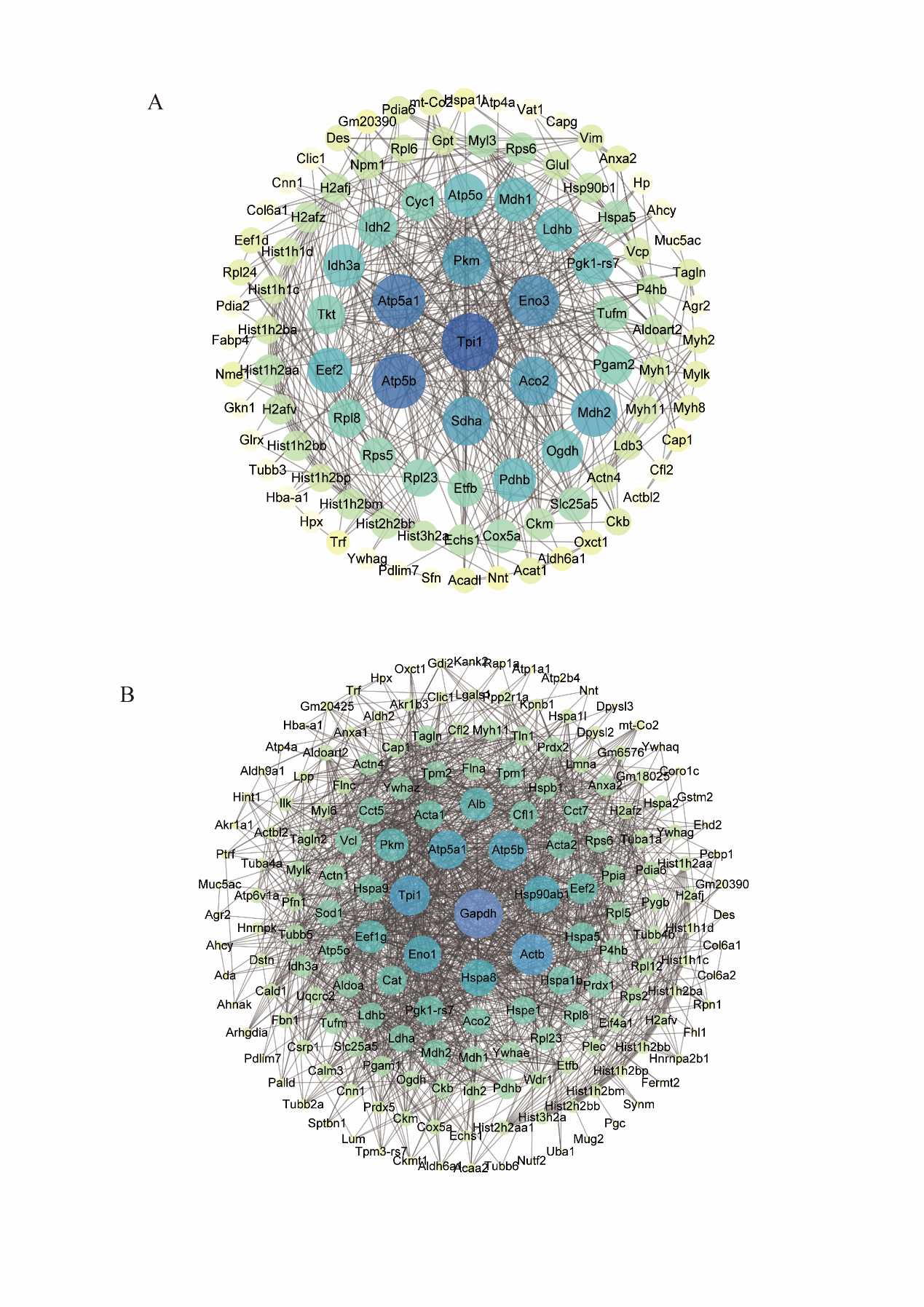


**Figure S2 |** Protein–protein interaction (PPI) networks of the intervened targets. **(A)** PPI networks of model group vs healthy group. **(B)** PPI networks of CR-treated group vs model group.


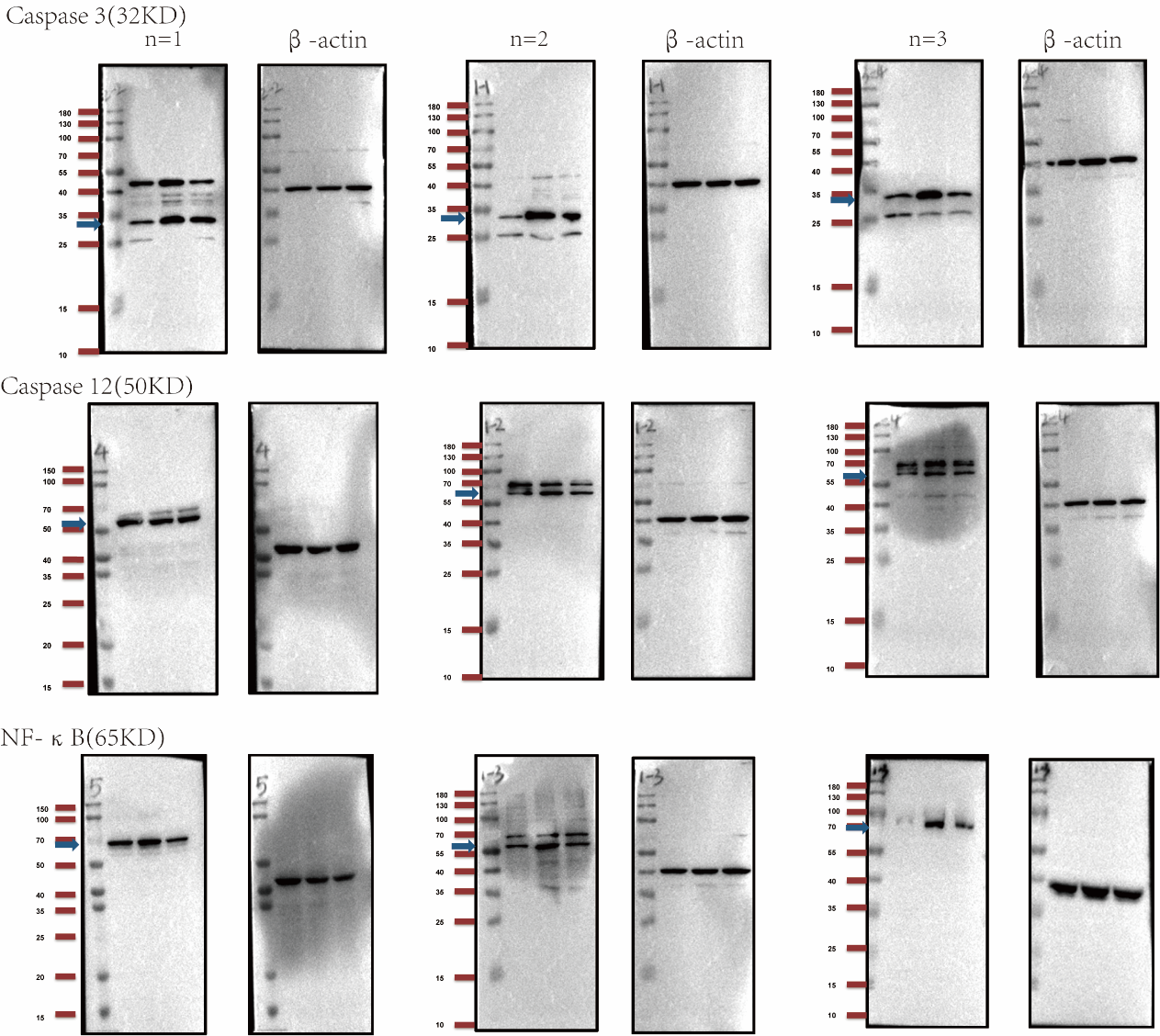


**Figure S3|** Western blots of Caspase3, Caspase12 and NF-κB in GES-1 cells
